# Supplementary material for: Native doublet microtubules from Tetrahymena thermophila reveal the importance of outer junction proteins
Source: Nat Commun. 2023 Apr 15;14:2168. doi: 10.1038/s41467-023-37868-0 (PMC10105768; doi:10.1038/s41467-023-37868-0)
Supplement: Supplementary file 1 — Supplementary Information [file 41467_2023_37868_MOESM1_ESM.pdf]

Supplementary Figures

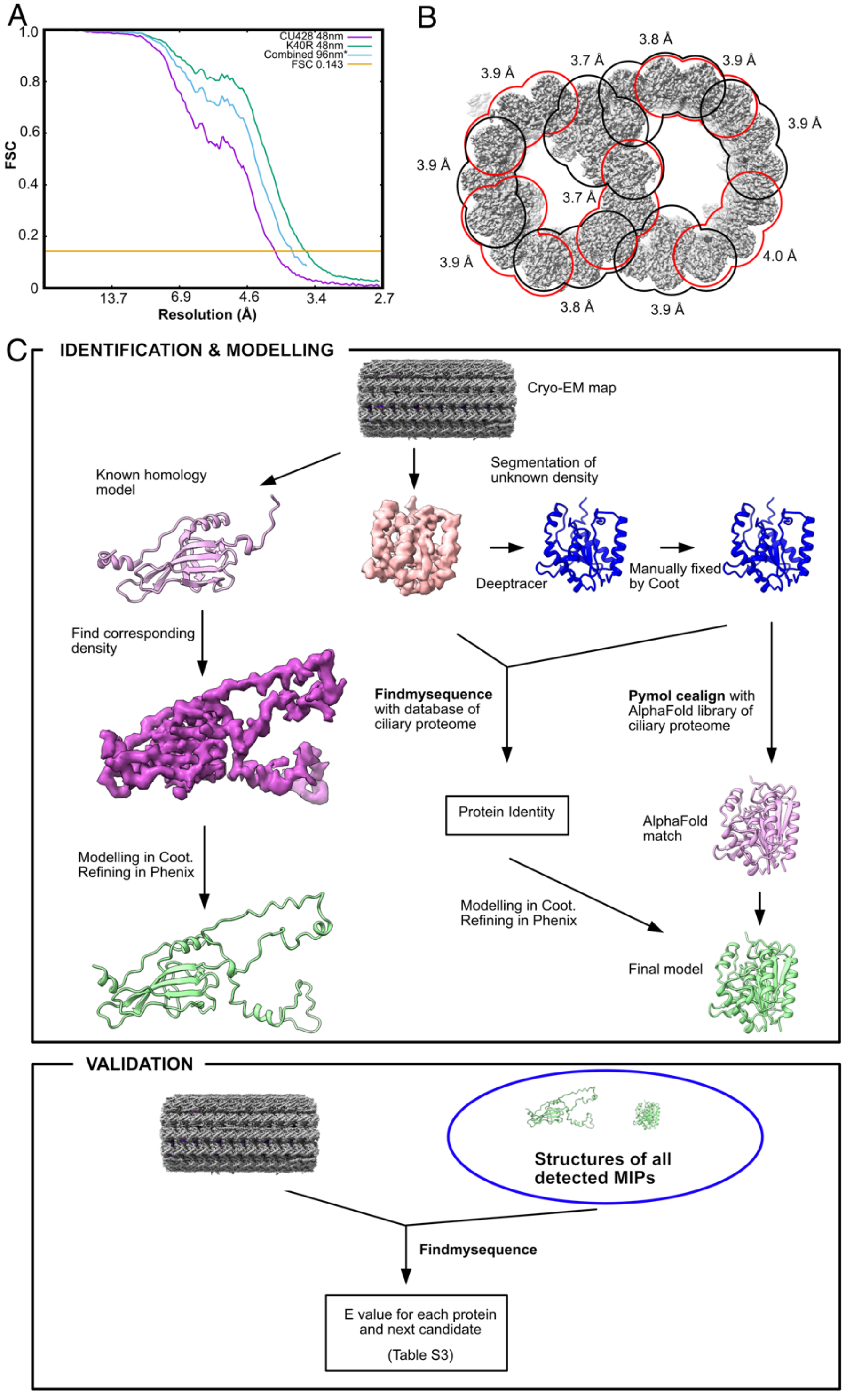

Supplementary Figure 1: Cryo-EM, modelling and validation. (A) Fourier Shell Correlation curves of the 48nm repeat maps of *WT* (*CU428*), *K40R* DMT and the 96-nm repeat map (both *CU428* and *K40R*) of the DMT. (B) The rough outline of masks used for local refinement of the DMT. A group of 2-3 PFs were contained in a mask (red or back outline) for focus-refinement. The resolutions of each group were noted next to the outline of the masks. (C) Strategy for the identification and modelling of proteins in the map (top). Strategy for the validation of the MIPs protein. See also Supplementary Table 4 for more information of validation.

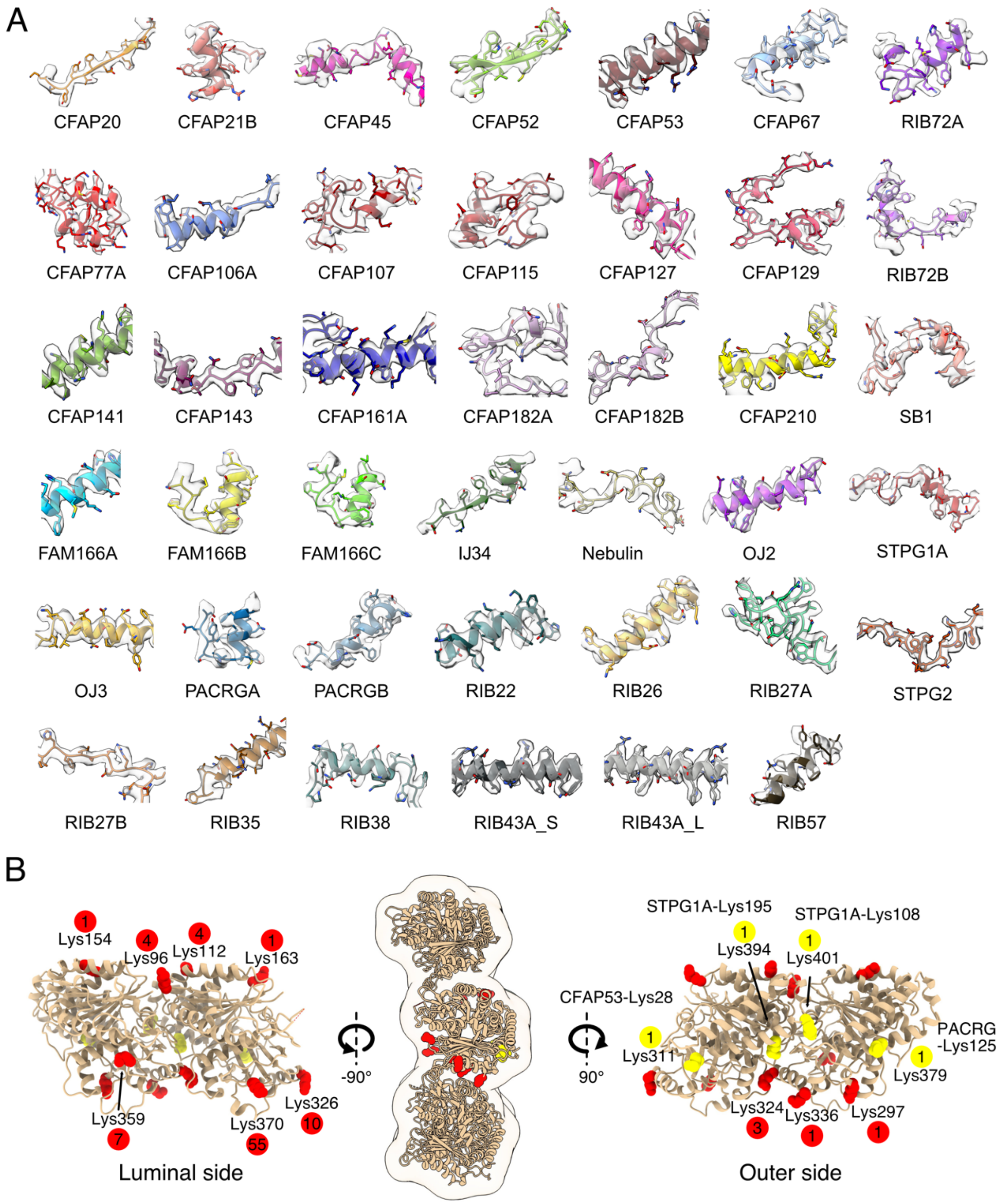

Supplementary Figure 2: Visualization of side-chain densities of MIPs and cross-links with tubulin. (A) Examples of cryo-EM maps and the respective atomic models of all *Tetrahymena* MIPs modeled in this study. (B) Mapping of mass spectrometry crosslinks from MIPs to corresponding tubulin residues. The numbers in the circle mean the number of crosslinks found from MIPs to the tubulin residues. While the four residues with crosslinks on the outer surface of tubulins (yellow) are linked to the outer domain of STPG1A, PACRGA and CFAP53, the rest of the crosslinks are strictly located to the luminal or the lateral regions.

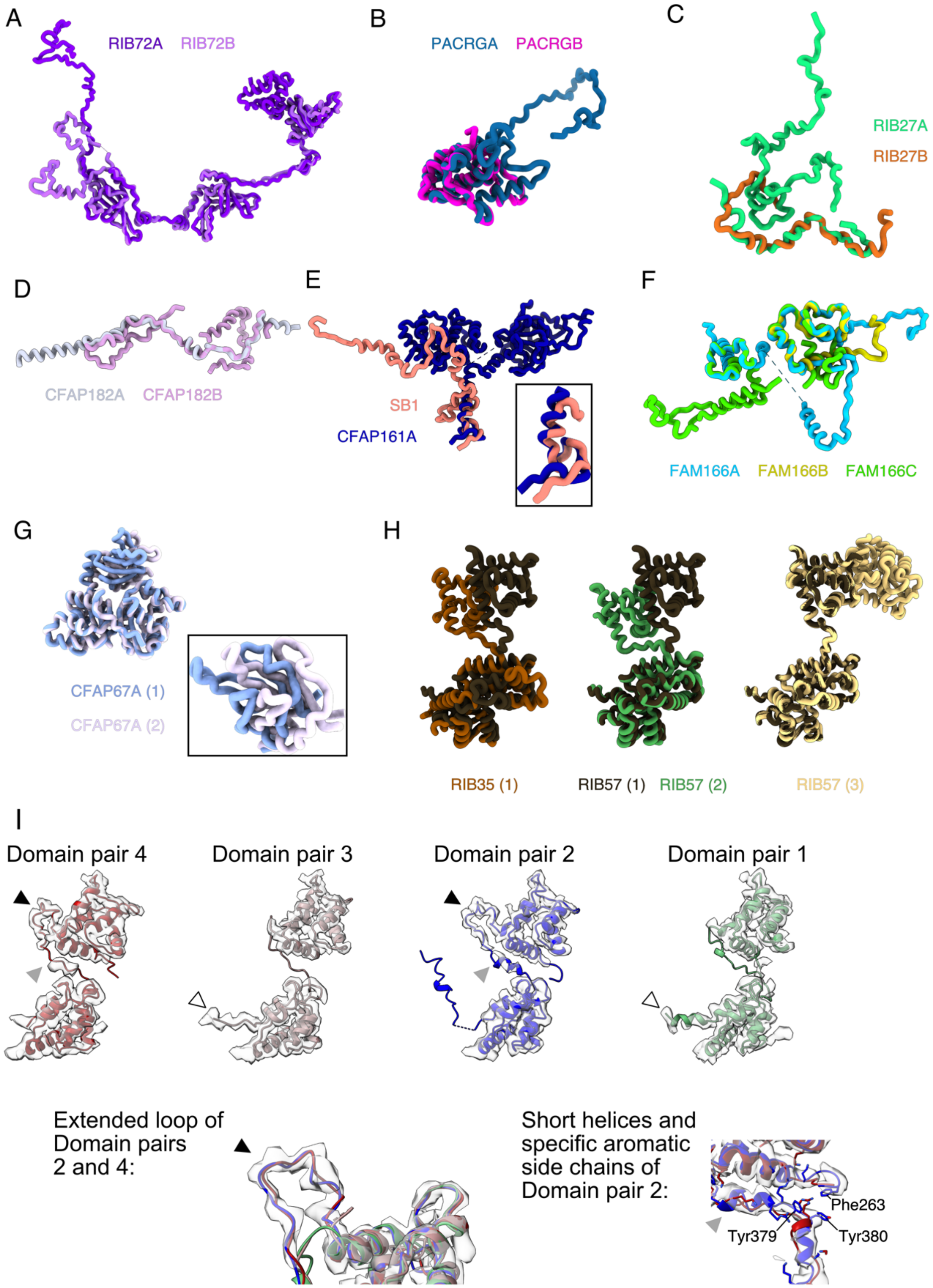

Supplementary Figure 3: Paralogs and unique conformations of *Tetrahymena* MIPs. (A-H) Structural comparisons of paralogs and unique conformations of *Tetrahymena* MIPs. (A) RIB72A and RIB72B. (B) PACRGA and PACRGB. (C) RIB27A and RIB27B. (D) CFAP182A and CFAP182B. (E) SB1 and CFAP161A. (F) FAM166A, FAM166B, and FAM166C. (G) CFAP67 repeats 1 and 2. (H) The EF-hand of repeat 1 of RIB57 is aligned with repeats 2 and 3, as well as with RIB35. (I) CFAP115 EF-hand domain pairs 1 and 3 are similar to each other, as are EF-hand domain pairs 2 and 4. However a short central helix and nearby aromatic side chains are unique to EF-hand domain pair 2.

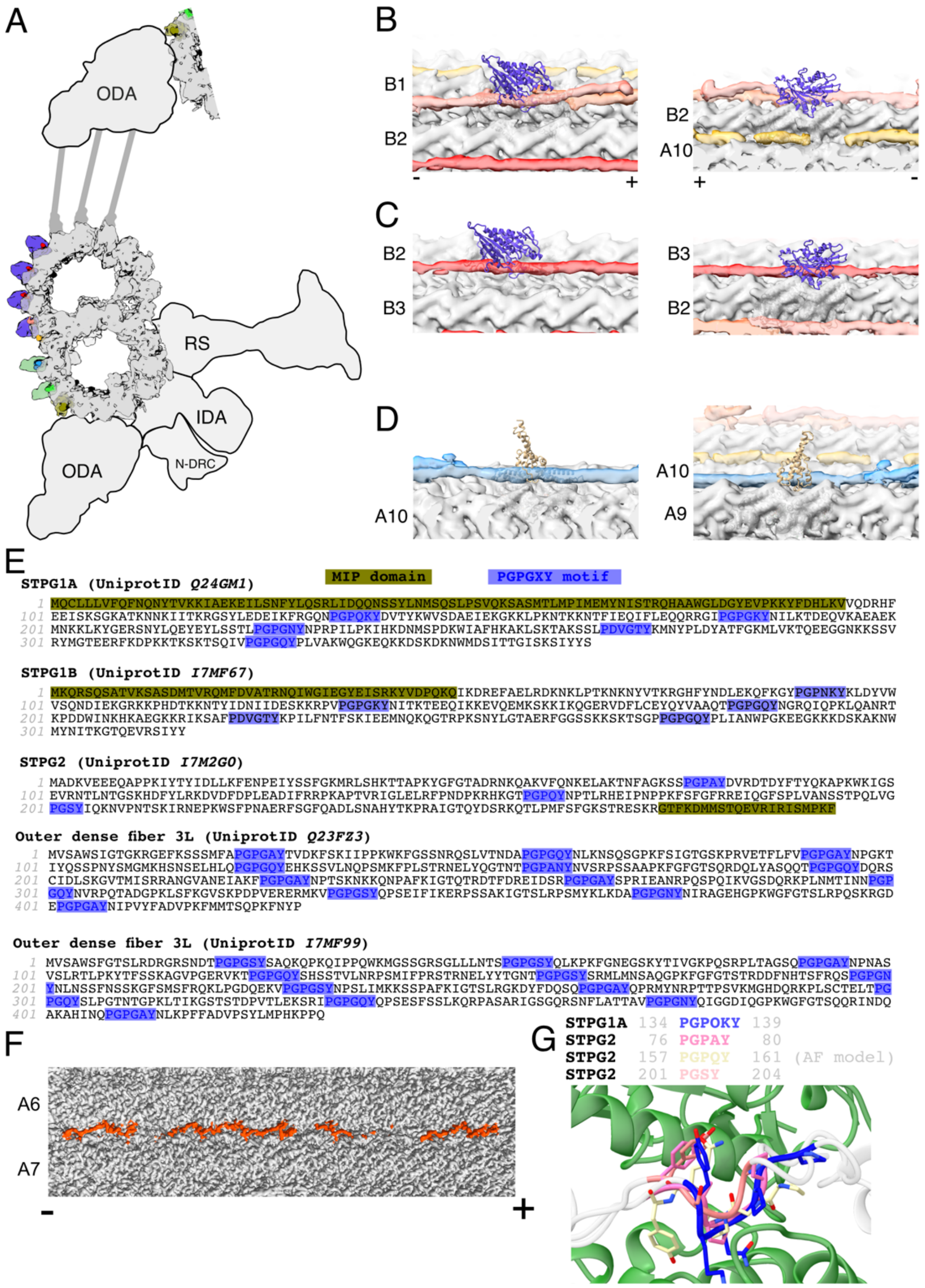

Supplementary Figure 4: The outer surface filament presents steric clashes with dynein and kinesin. (A) A cartoon of the DMT showing the putative regions for intraflagellar transport between A8-A10 and B1-B5. Violet densities: docking of kinesin microtubule-binding domain onto the B-tubule PFs, green densities: docking of dynein microtubule-binding domain onto the A8 and A9. ODA: Outer dynein arm; RS: Radial spoke; IDA: Inner dynein arm; N-DRC: Nexin-Dynein regulatory complex. (B-D) Docking of kinesin (PDB 6OJQ) on to the outer surface filament B1B2 (B) and B2B3 (C) showing a clear clash. (D) Docking of dynein-2 microtubule-binding domain (PDB 6RZB) onto the filament A9A10 showing the clear steric clash of microtubule-binding domain. (-) and (+) signs indicate the minus and plus ends of the microtubule respectively. (E) Amino acid sequences for STPG1A, STPG1B, STPG2, and two Outer dense fiber candidates. PG-rich repeat motifs (purple) and MIP domains (olive) are highlighted. (F) STPG2 is a filamentous protein that is woven between PFs A6-A7, present on the outer surface and the lumen. (G) PG-rich repeat motifs of STPG1A and STPG2 are structurally similar.

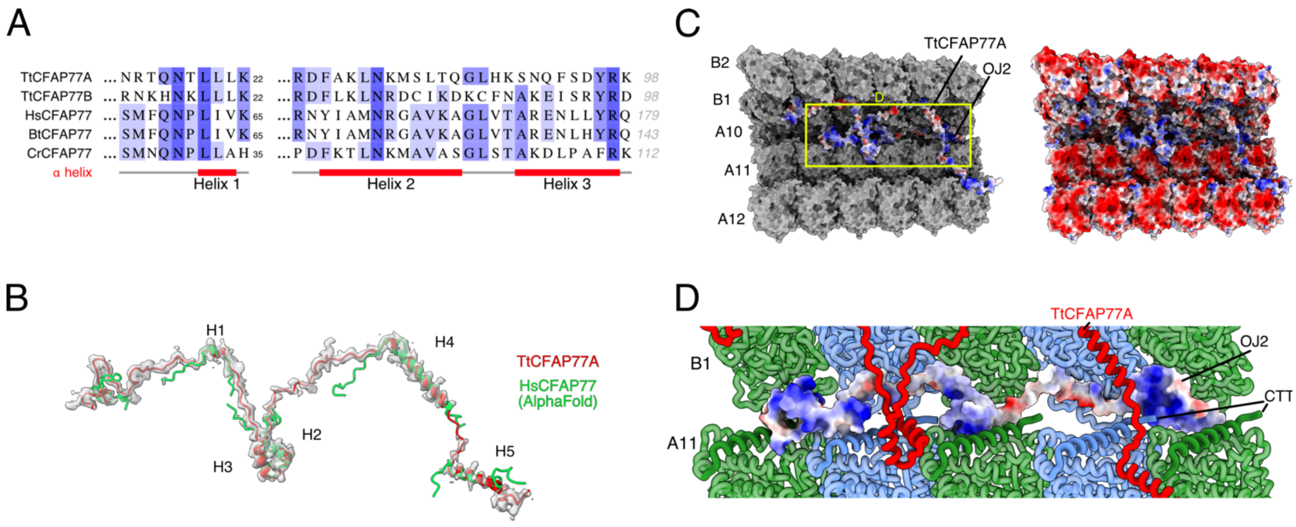

E

Average energy between A- and B-tubules		
	With OJ2	Without OJ2
With FAP77	-6.2604 ± 0.00408215	-6.05342 ± 0.0055639
Without FAP77	-7.18253 ± 0.00409181	-0.290828 ± 0.00524678
**Average energy between PFs B1 and B2**		
	With OJ2	Without OJ2
With FAP77	-30.2775 ± 0.00598608	-30.7353 ± 0.00608574
Without FAP77	-27.5101 ± 0.00881373	-26.6082 ± 0.00922062

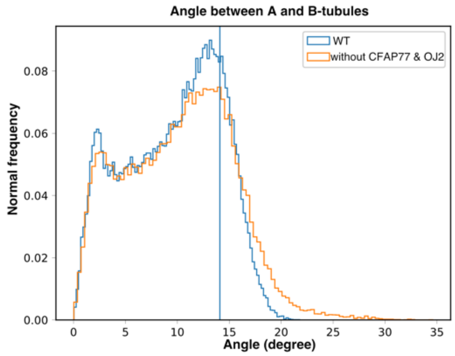

Supplementary Figure 5: Conservation of CFAP77. (A) Multiple sequence alignment of a segment of CFAP77. Human (Hs), bovine (Bt), and *Chlamydomonas* (Cr) sequences as well as two orthologs of *Tetrahymena* (Tt) were included. (B) Cryo-EM model of *Tetrahymena* TtCFAP77A. Folded regions of the AlphaFold2 model of human CFAP77 (green) were fitted into the map used to model the *Tetrahymena* protein. (C, D) Electrostatic potential of the outer junction proteins TtCFAP77 and OJ2 (C). Electropositive grooves of OJ2 are proximal to β-tubulin C-termini of PF A11 (D). (E) Stability of the outer junction in the presence and absence of CFAP77 and OJ2 based on coarse-grained molecular dynamics simulations. (F) Frequency of angular elasticity for A- and B-tubules from our MD simulations.

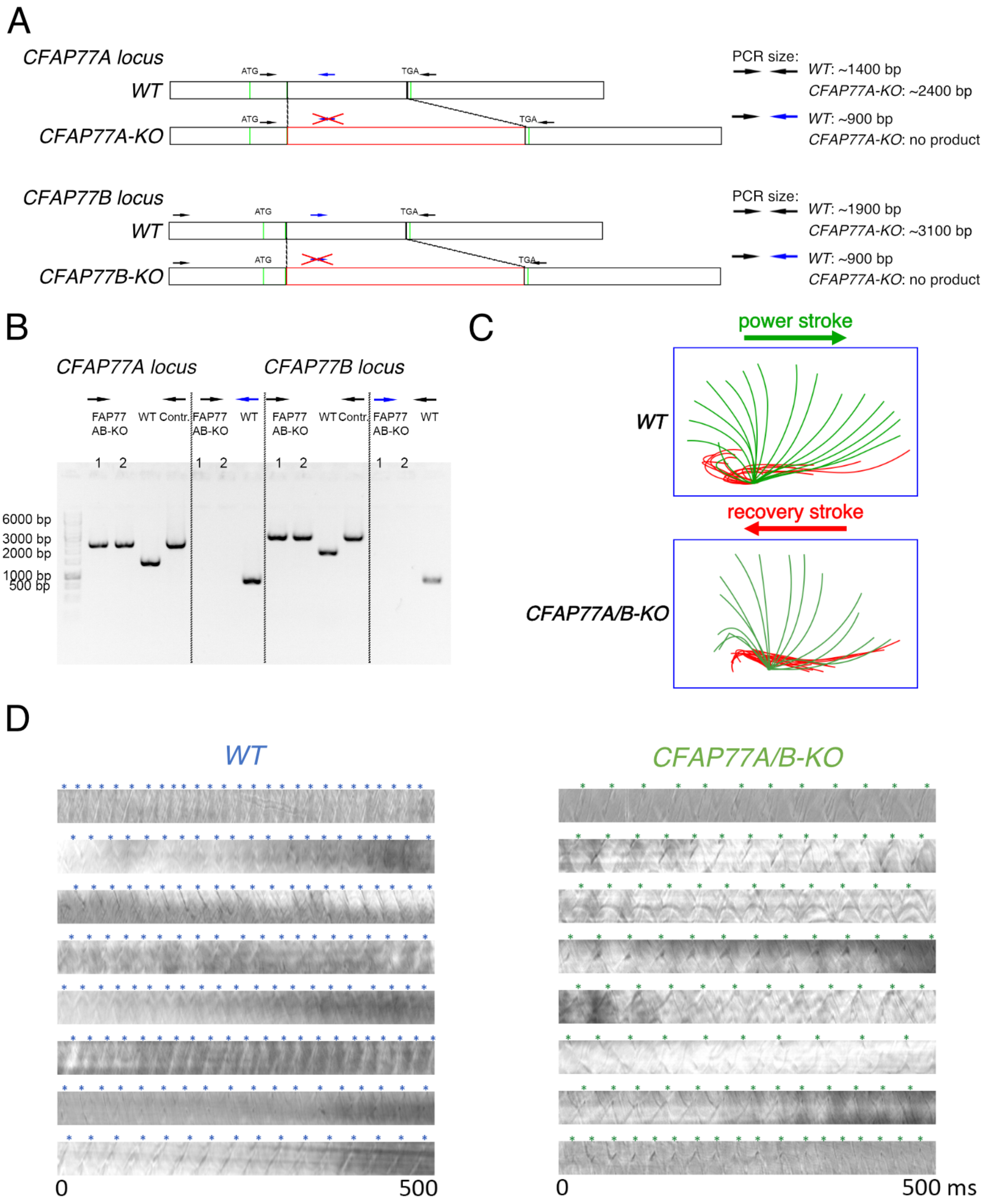

Supplementary Figure 6: Deletion of two paralogs, CFAP77A and CFAP77B reduces cilia beating frequency. (A) Homologous recombination methodology for *CFAP77A* and *CFAP77B* knockout strains. (B) PCR-based confirmation of the gene knockout. (C) Lack of both CFAP77 paralogs does not apparently alter cilia beating amplitude and waveform. (D) Examples of kymographs of cilia motility, generated from each movie in ImageJ. Each wave peak (*) on a kymograph corresponds to cilia passing through the drawn line. Source data are provided as a Source Data file for (B).

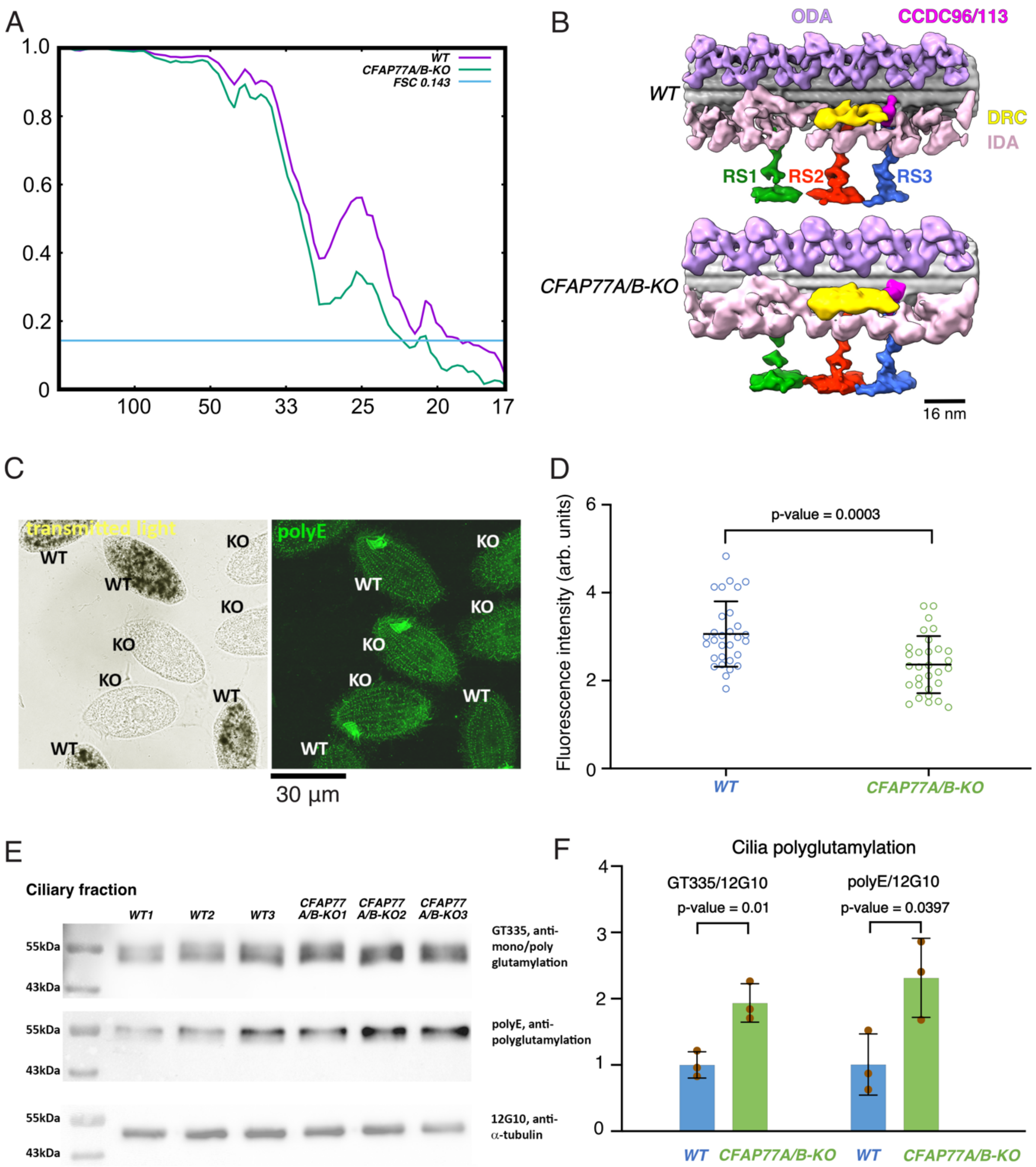

Supplementary Figure 7: **Cilia in the *CFAP77A/B-KO* mutant have a slightly higher level of tubulin glutamylation.** (A) Fourier Shell Correlation curves showing the determined resolution of *WT (CU-428)* and *CFAP77A/B-KO* 96-nm subtomogram averaged maps. (B) The 96-nm subtomogram averaged maps of *WT (CU-428)* and *CFAP77A/B-KO* show no abnormalities. (C) Phase-contrast (left) and immunofluorescence (right) of a mixed population of WT and *CFAP77A/B-KO* mutant cells stained with polyE antibodies detecting long glutamyl side chains (polyglutamylation). Note that WT cells were fed with India Ink and thus contain dark food vacuoles enabling their identification in the population of mixed cells. (D) Graph showing the corresponding quantitative immunofluorescence analyses of the average pixel intensity of axoneme region in mixed and processed side–by–side population of WT and mutant cells (n = 30 cells for each WT and *CFAP77A/B-KO* from one experiment). Fluorescence intensity is measured in arbitrary units. (E, F) Western blot (E) and densitometric analyses (graph, F) of the levels of tubulin glutamylation of ciliary tubulin in WT and *CFAP77A/B-KO* cells. The level of

tubulin was shown using anti-α-tubulin 12G10 antibodies, and levels of tubulin glutamylation were detected using GT335 (left) or polyE antibodies (right) (n = 3 biological replicates for WT and *CFAP77A/B-KO*). Data are presented as mean values +/- standard deviation in (D and F). Experiment is done one time for (C-F). Two-sided t-test was done for (D and F). Source data are provided as a Source Data file for (D-F).

Supplementary Tables

Supplementary Table 1: Conserved MIPs in *Tetrahymena, Chlamydomonas* and Bovine cilia. (*: proteins not modelled in this study; IJ: Inner junction, OJ: Outer junction, Ribbon: PFs A10-A13).

Region	*Tetrahymena*	*Chlamydomonas*	Bovine	Reference (cilia)	Reference (MIP)
A-tubule	TtFAM166A	-	FAM166A	[1]	[2], this study
A-tubule	TtFAM166B	-	FAM166B	[3]	[4]
A-tubule	TtFAM166C	-	FAM166C	[5]	[5]
A-tubule	TtCFAP21A	FAP21	CFAP21	[6]	[6]
A-tubule	TtCFAP21B*				
A-tubule	TtCFAP53	FAP53	CFAP53	[6]	[7]
A-tubule	TtCFAP67	FAP67	NME7	[6]	[7]
A-tubule	-	FAP68	CFAP68	[6]	[7]
A-tubule	TtCFAP115	FAP115		[6]	[7]
A-tubule	TtCFAP127	FAP127	MNS1	[6]	[7]
A-tubule	TtCFAP129	FAP129		[6]	[7]
A-tubule	TtCFAP182A	FAP182	PIERCE1	[6]	[7]
A-tubule	TtCFAP182B		PIERCE2		
B-tubule	TtCFAP45	FAP45	CFAP45	[6]	[8]
B-tubule	-	FAP90	CFAP90	[6]	[7]
B-tubule	TtCFAP112A	FAP112		[6]	[7]
B-tubule	TtCFAP112B*				
B-tubule	-	FAP144	FAM183A	[6]	[5]
B-tubule	TtCFAP210	FAP210	CFAP210	[6]	[7]
IJ	TtCFAP20	FAP20	CFAP20	[6]	[9]
IJ	TtCFAP52A	FAP52	CFAP52	[6]	[8]
IJ	TtCFAP52B*				
IJ	TtCFAP52C*				
IJ	-	FAP126	FLTP	[6]	[7]
IJ	-	FAP276	CFAP276	[7]	[7]
IJ	TtPACRGA	PACRG	PACRG	[10]	[11]
IJ	TtPACRGB				
IJ	TtPACRGC*				
OJ	TtCFAP77A	FAP77	CFAP77	[6]	[5]
OJ	TtCFAP77B*				
Ribbon	-	FAP95	CFAP95	[6]	[7]
Ribbon	TtCFAP106A	FAP106	ENKUR	[6]	[7]
Ribbon	TtCFAP106B*				
Ribbon	TtCFAP106C*				
Ribbon	TtCFAP107	FAP107	CFAP107	[6]	[7]
Ribbon	TtCFAP141	FAP141	CFAP141	[6]	[7]
Ribbon	TtCFAP143	FAP143	SPAG8	[6]	[7]
Ribbon	TtCFAP161A	FAP161	CFAP161	[6]	[7]
Ribbon	TtCFAP161B*				

Ribbon	TtRIB43a_S	RIB43a	RIBC2	12	13
Ribbon	TtRIB43a_L				
Ribbon	TtRIB72A	RIB72	EFHC1	14,15	16
	TtRIB72B		EFHC2		

Supplementary Table 2: Species-specific MIPs in *Tetrahymena, Chlamydomonas* and Bovine cilia.

Tetrahymena	Reference (cilia)	Chlamydomonas	Reference (cilia)	Bovine	Reference (cilia)
TtB3B4_fMIP	This study	FAP68	[6]	EFCAB6	[4,17]
TtB4B5_fMIP	This study	FAP85	[18]	TEKTIN 1	[4,19,20]
TtB5B6_fMIP	This study	FAP90	[6]	TEKTIN 2	[4,19,20]
TtIJ34	This study	FAP166	[6]	TEKTIN 3	[4,19,20]
TtOJ2	This study	FAP222	[6]	TEKTIN 4	[4,19,20]
TtOJ3	This study	FAP252	[6,21]	TEKTIP1	[4]
TtRIB22	This study	FAP273	[22]		
TtRIB26	This study	FAP363	[7]		
TtRIB27A	This study	RIB21	[7]		
TtRIB27B	This study	RIB30	[7]		
TtRIB35	This study				
TtRIB38	This study				
TtRIB57	This study				
SB1	This study				
STPG1A	This study				
STPG1B	This study				
STPG2	This study				
Nebulin	This study				

Supplementary Table 3: UniProt ID and Gene name of the *Tetrahymena* MIPs in this study. (*: the proteins are found from mass spectrometry of *K40R*, *RIB72A/B* and *RIB72B-KO* mutants).

MIP	UniProt ID	Gene name	emPAI score
CFAP20	Q22NU3	TTHERM_00418580	20.72
CFAP21A	I7MK20	TTHERM_00340080	0.66
CFAP21B	I7MLS4	TTHERM_00494280	3.03
CFAP45	W7XCX2	TTHERM_001164064	3.51
CFAP52A	Q22ZH2	TTHERM_01094880	14.66
CFAP52B	I7MJ23	TTHERM_00836640	1.78
CFAP52C	Q24C92	TTHERM_00695800	1.44
CFAP53	Q23YQ8	TTHERM_01207630	3.53
CFAP67	W7XGD1	TTHERM_000372529	5.00
CFAP77A	Q22WR6	TTHERM_00974270	4.87
CFAP77B	Q239Q2	TTHERM_01190430	2.31
CFAP106A	I7M279	TTHERM_00137550	6.18
CFAP106B	I7LU20	TTHERM_00370750	3.67
CFAP106C	Q238Q9	TTHERM_00454070	0.13
CFAP107	Q237T1	TTHERM_00320040	0.98
CFAP112A (B3B4_fMIP)	Q23A15	TTHERM_00884620	1.86
CFAP112B (B5B6_fMIP)	I7MEK6	TTHERM_00192030	2.79
CFAP115	Q23KF9	TTHERM_00193760	14.13
CFAP127	I7LV70	TTHERM_00338110	3.66
CFAP129	I7M9I4	TTHERM_00565590	9.30
CFAP141	A4VCU8	TTHERM_00242188	0.41
CFAP143	A4VD56	TTHERM_00502359	5.78
CFAP161A	Q22WJ6	TTHERM_00155380	4.43
CFAP161B	I7M8Z8	TTHERM_00219350	4.73
CFAP182A	Q24BV4	TTHERM_01049330	4.20
CFAP182B	I7MLW3	TTHERM_00535210	*
CFAP210	Q23EX8	TTHERM_00643530	2.73
FAM166A	Q238X3	TTHERM_00449470	32.46
FAM166B	Q235M9	TTHERM_01052990	6.31
FAM166C	Q22B75	TTHERM_01109800	3.15
IJ34	I7M9T0	TTHERM_00348390	5.50
Nebulin	Q231B6	TTHERM_00433410	1.12
OJ2	Q236L2	TTHERM_00086850	10.66
OJ3	Unknown	Unknown	-
PACRGA	I7MLV6	TTHERM_00446290	19.16
PACRGB	I7M317	TTHERM_00499570	30.24
PACRGC	I7M312	TTHERM_00499310	*
RIB22	I7LT67	TTHERM_00691900	6.14
RIB26	Q232I6	TTHERM_00594110	2.33
RIB27A (Tex36A)	I7LUL4	TTHERM_00522810	3.01
RIB27B	Q22CT6	TTHERM_01016050	7.95
RIB35	I7ME81	TTHERM_00267880	12.44
RIB38 (Tex36B)	Q23JL9	TTHERM_00758860	7.92
RIB43A_L	Q240R7	TTHERM_00624660	1.69
RIB43A_S	A4VDZ5	TTHERM_00641119	4.29
RIB57	I7ME23	TTHERM_00406590	26.04
RIB72A	I7M0S7	TTHERM_00143690	13.16
RIB72B	I7MCU1	TTHERM_00584850	12.40
SB1 (Seam Binding 1)	Q231B2	TTHERM_00433450	3.08
STPG1A	Q24GM1	TTHERM_00724700	2.19
STPG1B	I7MF67	TTHERM_00134870	1.05
STPG2	I7M2G0	TTHERM_00218910	4.82

Supplementary Table 4: Identification of *Tetrahymena* proteins based on side chain fitting of our cryo-EM map. The E-value is reported from *FindMySequence* search of C-alpha backbone against the *K40R* cryo-EM map with proteins in the ciliome.

MIP	UniProt ID	E-value	2nd match	E-value (2nd)	3rd match	E-value (3rd)
CFAP20	Q22NU3	2.60E-52	Q22SL3	6.70E-03		
CFAP21B	I7MLS4	6.30E-80	I7MK20 (CFAP21A)	7.90E-05		
CFAP45	W7XCX2	7.10E-10				
CFAP52A	Q22ZH2	8.40E-164	I7MJ23 (CFAP52B)	7.10E-21	Q24C92 (CFAP52C)	4.00E-13
CFAP53	Q23YQ8	3.00E-60	A4VD76	1.20E-01		
CFAP67	W7XGD1	5.20E-126	I7M7Q3	2.10E-85	Q23U54	6.00E-09
CFAP77A	Q22WR6	4.90E-31	Q239Q2 (CFAP77B)	6.30E-21		
CFAP106A	I7M279	5.20E-53	I7LU20 (CFAP106B)	4.60E-23	Q238Q9 (CFAP106C)	7.30E-21
CFAP107	Q237T1	7.50E-20				
CFAP112A (B3B4_fMIP)	Q23A15	1.70E-06				
CFAP112B (B5B6_fMIP)	I7MEK6	8.20E-06				
CFAP115	Q23KF9	9.20E-105	W7XC24	1.40E-04		
CFAP127	I7LV70	1.40E-37				
CFAP129	I7M9I4	1.40E-18				
CFAP141	A4VCU8	2.70E-27				
CFAP143	A4VD56	1.70E-39				
CFAP161A	Q22WJ6	3.40E-95	I7M8Z8 (CFAP161B)	5.10E-41	I7ME23 (RIB57)	1.60E-06
CFAP182A	Q24BV4	1.80E-17				
CFAP182B	I7MLW3	6.90E-20				
CFAP210	Q23EX8	2.10E-22	W7XDG5	2.10E-03	Q22GH8	2.20E-02
FAM166A	Q238X3	6.80E-21	Q235M9 (FAM166B)	4.70E-05		
FAM166B	Q235M9	1.90E-20	Q238X3 (FAM166A)	1.20E-06		
FAM166C	Q22B75	1.10E-43	Q238X3 (FAM166A)	1.70E-01		
IJ34	I7M9T0	1.50E-95				
Nebulin	Q231B6	4.50E-06				
OJ2	Q236L2	1.90E-37	I7MAL9	5.70E-14	Q24DL2	5.50E-11
PACRGA	I7MLV6	5.10E-72	I7M312 (PACRGC)	3.10E-49		
PACRGB	I7M317	3.80E-57	I7MLV6 (PACRGA)	2.10E-39		
RIB22	I7LT67	3.80E-54	I7ME23 (RIB57)	1.20E-10	I7M8Z8 (CFAP161B)	5.00E-10
RIB26	Q232I6	2.20E-83				
RIB27A	I7LUL4	7.40E-23	Q22CT6 (RIB27B)	5.10E-11	Q22B75 (FAM166C)	1.10E-01
RIB27B	Q22CT6	1.30E-04	I7LUL4 (RIB27)	1.40E-02		
RIB35	I7ME81	1.20E-73	I7LT67 (RIB22)	1.60E-09	I7M8Z8 (CFAP161B)	9.60E-09

RIB38/Tex36B	Q23JL9	1.00E-76	Q233Y0	4.80E-25		
RIB43A_S	A4VDZ5	9.50E-41	Q240R7 (RIB43A_L)	8.50E-08		
RIB43A_L	Q240R7	3.30E-64	A4VDZ5 (RIB43A_S)	3.60E-04	W7XC77 (PG-rich)	4.00E-04
RIB57	I7ME23	1.30E-127	I7LT67 (RIB22)	5.50E-08	I7M8Z8 (CFAP161B)	9.80E-07
RIB72A	I7M0S7	1.10E-204	I7MCU1 (RIB72B)	2.30E-50		
RIB72B	I7MCU1	3.00E-164	I7M0S7 (RIB72A)	6.00E-53		
SB1 (SeamBinding1)	Q231B2	3.90E-31				
STPG1A	Q24GM1	2.80E-28	I7MF67 (STPG1B)	7.60E-17		
STPG2	I7M2G0	6.70E-16				

Supplementary Table 5: *In situ* crosslinks between MIPs and tubulins in *Tetrahymena*.

Protein 1	AA Number	Protein 2	AA Number	Protein 1	AA Number	Protein 2	AA Number
TUBA	370	CFAP45	422	TUBA	370	NEBULIN	449
TUBA	370	CFAP45	364	TUBA	370	NEBULIN	436
TUBA	370	CFAP45	308	TUBA	370	FAM166A	196
TUBA	370	CFAP45	209	TUBA	96	RIB27A/TEX36A	228
TUBA	370	CFAP45	429	TUBA	326	IJ34	114
TUBA	370	CFAP45	314	TUBA	326	STPG2	276
TUBA	370	CFAP115	544	TUBA	326	STPG2	151
TUBA	370	CFAP115	492	TUBA	326	STPG2	154
TUBA	370	CFAP115	910	TUBA	370	CFAP143	49
TUBA	326	CFAP161A	253	TUBA	370	CFAP143	47
TUBA	326	CFAP53	314	TUBA	370	RIB38/Tex36B	223
TUBA	311	CFAP53	28	TUBA	370	RIB38/Tex36B	35
TUBA	370	CFAP53	370	TUBA	96	CFAP21A	184
TUBA	370	CFAP210	278	TUBA	370	CFAP21A	7
TUBA	370	CFAP210	326	TUBA	370	CFAP107	161
TUBA	370	CFAP210	268	TUBA	370	CFAP107	146
TUBA	370	CFAP210	220	TUBA	370	CFAP107	114
TUBA	370	CFAP52	442	TUBA	370	CFAP21B	6
TUBA	370	CFAP52	183	TUBA	401	STPG1A	108
TUBA	370	CFAP112B	451	TUBA	96	STPG1A	296
TUBA	370	CFAP112B	297	TUBA	394	STPG1A	195
TUBA	370	CFAP112B	287	TUBA	112	STPG1A	296
TUBA	112	CFAP106A	150	TUBA	96	STPG1A	297
TUBA	370	CFAP112A	264	TUBA	370	FAM166C	209
TUBA	370	CFAP112A	428	TUBA	370	CFAP129	131
TUBA	370	CFAP112A	210	TUBA	370	SB1	98
TUBA	370	CFAP112A	369	TUBA	326	RIB27B	168
TUBA	370	CFAP112A	254	TUBA	370	RIB43a_L	127
TUBA	370	CFAP112A	318	TUBA	163	PACRGB	86
TUBA	370	CFAP112A	475	TUBA	326	ODF3L-1 (I7MF99)	330
TUBA	112	CFAP112A	13	TUBA	326	ODF3L-2 (Q23FZ3)	322
TUBA	112	CFAP112A	5	TUBA	326	ODF3L-2 (Q23FZ3)	367
TUBA	370	CFAP112A	380	TUBA	326	ODF3L-2 (Q23FZ3)	397
TUBA	370	RIB57	22	TUBA	326	ODF3L-2 (Q23FZ3)	80
TUBA	370	RIB57	253	TUBB	359	CFAP45	291
TUBA	326	RIB72B	239	TUBB	359	CFAP45	237
TUBA	370	RIB72B	223	TUBB	359	CFAP115	748
TUBA	326	RIB72B	401	TUBB	359	CFAP115	521
TUBA	370	RIB72B	23	TUBB	297	CFAP161A	253
TUBA	326	RIB72A	297	TUBB	359	CFAP53	302
TUBA	370	NEBULIN	12	TUBB	359	CFAP210	199
TUBA	370	NEBULIN	356	TUBB	379	PACRGA	125
TUBA	370	NEBULIN	403	TUBB	154	PACRGA	17
TUBA	370	NEBULIN	442	TUBB	359	RIB72B	219
TUBA	370	NEBULIN	148	TUBB	324	STPG1A	189
TUBA	370	NEBULIN	189	TUBB	324	CFAP129	254
TUBA	370	NEBULIN	60	TUBB	324	SB1	104
TUBA	370	NEBULIN	194	TUBB	336	PACRGB	38

Supplementary Table 6: Mass spectrometry analysis of wild type (WT), *RIB72B* and *RIB72A/B* knockout mutants showing the missing proteins. (Only proteins with quantitative value > 1 are shown). Source data are provided as a Source Data file.

**UniprotID**	***WT (CU428)***			***RIB72B-KO***			***RIB72A/B-KO***		
	**S1**	**S2**	**S3**	**S1**	**S2**	**S3**	**S1**	**S2**	**S3**
I7MCU1 (**RIB72B**)	217.4	199.6	212.8	0.0	0.0	0.0	0.0	0.0	0.0
I7M0S7 (**RIB72A**)	172.9	189.0	181.7	246.0	245.9	242.0	0.0	0.0	0.0
Q23KF9 (**FAP115**)	300.3	313.5	297.0	294.5	281.9	281.9	0.0	0.0	0.0
Q238X3 (**FAM166C**)	101.3	102.4	115.2	84.5	89.0	102.7	0.0	0.0	0.0
Q235M9 (**FAM166B**)	35.8	34.4	44.3	33.8	39.2	38.8	0.0	0.0	0.0
I7LT67 (**RIB22**)	31.4	30.9	37.2	16.9	21.2	21.7	0.0	0.0	0.0
I7LUL4 (**RIB27A**)	28.8	20.3	31.9	13.7	27.6	30.8	0.0	0.0	0.0
Q22CT6 (**RIB27B**)	27.9	36.2	39.0	1.1	4.2	3.4	0.0	0.0	0.0
W7XC77 (PG-rich)	13.1	11.5	16.0	7.4	8.5	12.6	0.0	0.0	0.0
I7LV80	10.5	12.4	9.8	0.0	0.0	1.1	0.0	0.0	0.0
Q22TY0	9.6	8.8	8.9	0.0	0.0	0.0	0.0	0.0	0.0
I7LVP2	8.7	11.5	9.8	0.0	1.1	2.3	0.0	0.0	0.0
Q231F9	5.2	1.8	7.1	1.1	0.0	1.1	0.0	0.0	0.0
Q22CT4	5.2	4.4	3.5	0.0	0.0	1.1	0.0	0.0	0.0
I7M2F8	2.6	4.4	2.7	0.0	41.3	3.4	0.0	0.0	0.0
I7M6E1	2.6	3.5	1.8	0.0	0.0	0.0	0.0	0.0	0.0
Q22KN2	1.7	2.6	1.8	0.0	0.0	0.0	0.0	0.0	0.0
Q22HI8	1.7	0.9	0.9	0.0	0.0	0.0	0.0	0.0	0.0
I7MDS6	1.7	0.9	1.8	0.0	0.0	0.0	0.0	0.0	0.0
I7LWU5	1.7	0.9	1.8	1.1	1.1	0.0	0.0	0.0	0.0
Q22S92	1.7	0.0	0.9	0.0	0.0	0.0	0.0	0.0	0.0

Supplementary Table 7: Primers used to engineer *Tetrahymena CFAP77* paralogs mutant cells. Sequence recognized by restriction endonuclease in bold, ATG or TGA are underlined.

Name	Sequence	Amplified fragments
	**CFAP77A-3HA native locus expression**	
CFAP77A-3HA-cod-MluI-F	AAAT **ACGCGT** T ATG TCAAGAACTAGCACCGG	entire open reading frame (1.58 kb)
CFAP77A-3HA-cod-BamHI-R	AAAT **GGATCC** CTCTACAGCTTGGTTAGGAACT	
CFAP77A-3HA-3UTR-PstI-F	AAAT **CTGCAG** CTTTAGTGATTTGCCAGTTTTAAGATC	0.8 kb of a 3'UTR
CFAP77A-3HA-3UTR-XhoI-R	AAAT **CTCGAG** CATAACTCTTAATGAA AATTGAGAGATAGTTTC	transgene enabling expression of CFAP77A with C- tag in native locus
	**CFAP77B-3HA native locus expression**	
CFAP77B-3HA-cod-MluI-F	AAAT **ACGCGT** T ATG AATTTGAGTTAAGAAGGAGTTCT	entire open reading frame (1.26 kb)
CFAP77B-3HA-cod-BamHI-R	AAAT **GGATCC** TGCAGCATGTTCTGA GCTTTAATTAAGGAA	
CFAP77B-3HA-3UTR-PstI-F	AAAT **CTGCAG** AGAGTTATAATTCTTTG TTACACTTCTACAG	0.6 kb of a 3'UTR
CFAP77B-3HA-3UTR-XhoI-R	AAAT **CTCGAG** TTATCAGCAATCATCTCTTCTCTCT	transgene enabling expression of CFAP77B with C- tag in native locus
	**CFAP77A-KO**	
CFAP77A-KO-5-F-ApaI	AATT **GGGCCC** AAGAATCTATTTATTGATTTAACCATC	1 kb fragment encompassing 0.5 kb of the 5'UTR and 0.5 kb of the open reading frame, upstream to the deleted gene fragment (1 kb)
CFAP77A-KO-5-R-EcoRV-SmaI	AATT **CCCGGG** ATATCGGTATTTCCA AGTCATGCTAACTATTTA	
CFAP77A-KO-3-F-PstI	AATT **CTGCAG** CTATGATGCTAACGAAGTTCCTAAC	1.4 kb fragment encompassing 37 bp of the open reading frame and 1.4 kb of 3'UTR downstream to the deleted gene fragment
CFAP77A-KO-3-R-EcoRV-SacI	AATT **CCGCGG** ATATCTAGCTAAATATT TTCTGGAAACTTAAATATCTAC	Transgene to knockout *CFAP77A* gene
	**CFAP77B-KO**	
CFAP77B-KO-5-F-ApaI	AATT **GGGCCC** TTTTGCAAGTATGAATACTACTTTTTGTT	1 kb fragment encompassing 0.6 kb of the 5'UTR and 0.4 kb of the open reading frame, upstream to the deleted gene fragment (0.8 kb)
CFAP77B-KO-5-R-EcoRV-SmaI	AATT **CCCGGG** ATATCGCGATTGAGC TTTAAAAAATCTCTAG	
CFAP77B-KO-3-F-PstI	AATT **CTGCAG** TATTGTAAACGATTTCAA GTTAGAAAGATTT	1.9 kb fragment encompassing 130 bp of the open reading frame and 1.77 kb of 3'UTR downstream to the deleted gene fragment
FAP77B-KO-3-R-EcoRV-SacI	AATT **CCGCGG** ATATCATGAATTAAGATTTGTTGCCATAG	Transgene to knockout *CFAP77B* gene
	**KO verification**	
CFAP77A-KO-Check-F	AGCGAGAAGATTTATAGAATGAATTTT	
CFAP77A-KO-Check-R1	CCTATTCAATACGCTGTTAGAATTCTA	no PCR product in KO
CFAP77A-KO-Check-R2	AAAGGTGTTAGTTAGTTAGTTAGCTAG	
CFAP77B-KO-5-F-ApaI	AATT **GGGCCC** TTTTGCAAGTATGAATACTACTTTTTGTT	
CFAP77B-KO-check-F	CAAATGCTTTAATGCGAAGGTAAC	no PCR product in KO
CFAP77B-KO-check-R	GGAACTCCTCTTAGCTGTGA	

Supplementary References

1 Lehti, M. S., Kotaja, N. & Sironen, A. KIF3A is essential for sperm tail formation and manchette function. *Mol Cell Endocrinol* **377**, 44-55 (2013). https://doi.org:10.1016/j.mce.2013.06.030
2 Leung, M. R. *et al.* Unraveling the intricate microtubule inner protein networks that reinforce mammalian sperm flagella. *bioRxiv* (2022).
3 Tu, F., Sedzinski, J., Ma, Y., Marcotte, E. M. & Wallingford, J. B. Protein localization screening in vivo reveals novel regulators of multiciliated cell development and function. *J Cell Sci* **131** (2018). https://doi.org:10.1242/jcs.206565
4 Gui, M. *et al.* De novo identification of mammalian ciliary motility proteins using cryo-EM. *Cell* **184**, 5791-5806.e5719 (2021). https://doi.org:https://doi.org/10.1016/j.cell.2021.10.007
5 Gui, M. *et al.* SPACA9 is a lumenal protein of human ciliary singlet and doublet microtubules. *Proc Natl Acad Sci U S A* **119**, e2207605119 (2022). https://doi.org:10.1073/pnas.2207605119
6 Pazour, G. J., Agrin, N., Leszyk, J. & Witman, G. B. Proteomic analysis of a eukaryotic cilium. *J Cell Biol* **170**, 103-113 (2005). https://doi.org:10.1083/jcb.200504008
7 Ma, M. *et al.* Structure of the Decorated Ciliary Doublet Microtubule. *Cell* **179**, 909-922 e912 (2019). https://doi.org:10.1016/j.cell.2019.09.030
8 Owa, M. *et al.* Inner lumen proteins stabilize doublet microtubules in cilia and flagella. *Nat Commun* **10**, 1143 (2019). https://doi.org:10.1038/s41467-019-09051-x
9 Yanagisawa, H. A. *et al.* FAP20 is an inner junction protein of doublet microtubules essential for both the planar asymmetrical waveform and stability of flagella in Chlamydomonas. *Mol Biol Cell* **25**, 1472-1483 (2014). https://doi.org:10.1091/mbc.E13-08-0464
10 Dawe, H. R., Farr, H., Portman, N., Shaw, M. K. & Gull, K. The Parkin co-regulated gene product, PACRG, is an evolutionarily conserved axonemal protein that functions in outer-doublet microtubule morphogenesis. *J Cell Sci* **118**, 5421-5430 (2005). https://doi.org:10.1242/jcs.02659
11 Dymek, E. E. *et al.* PACRG and FAP20 form the inner junction of axonemal doublet microtubules and regulate ciliary motility. *Mol Biol Cell* **30**, 1805-1816 (2019). https://doi.org:10.1091/mbc.E19-01-0063
12 Norrander, J. M., deCathelineau, A. M., Brown, J. A., Porter, M. E. & Linck, R. W. The Rib43a protein is associated with forming the specialized protofilament ribbons of flagellar microtubules in Chlamydomonas. *Mol Biol Cell* **11**, 201-215 (2000).
13 Ichikawa, M. *et al.* Tubulin lattice in cilia is in a stressed form regulated by microtubule inner proteins. *Proc Natl Acad Sci U S A* **116**, 19930-19938 (2019). https://doi.org:10.1073/pnas.1911119116
14 Ikeda, K. *et al.* Rib72, a conserved protein associated with the ribbon compartment of flagellar A-microtubules and potentially involved in the linkage between outer doublet microtubules. *J Biol Chem* **278**, 7725-7734 (2003). https://doi.org:10.1074/jbc.M210751200
15 Patel-King, R. S., Benashski, S. E. & King, S. M. A bipartite Ca2+-regulated nucleoside-diphosphate kinase system within the Chlamydomonas flagellum. The regulatory subunit p72. *J Biol Chem* **277**, 34271-34279 (2002). https://doi.org:10.1074/jbc.M204137200
16 Stoddard, D. *et al.* Tetrahymena RIB72A and RIB72B are microtubule inner proteins in the ciliary doublet microtubules. *Mol Biol Cell* **29**, 2566-2577 (2018). https://doi.org:10.1091/mbc.E18-06-0405
17 Patir, A. *et al.* The transcriptional signature associated with human motile cilia. *Sci Rep* **10**, 10814 (2020). https://doi.org:10.1038/s41598-020-66453-4
18 Kirima, J. & Oiwa, K. Flagellar-associated Protein FAP85 Is a Microtubule Inner Protein That Stabilizes Microtubules. *Cell Struct Funct* **43**, 1-14 (2018). https://doi.org:10.1247/csf.17023
19 Pirner, M. A. & Linck, R. W. Tektins are heterodimeric polymers in flagellar microtubules with axial periodicities matching the tubulin lattice. *J Biol Chem* **269**, 31800-31806 (1994).

20 Linck, R. W. Flagellar doublet microtubules: fractionation of minor components and alpha-tubulin from specific regions of the A-tubule. *J Cell Sci* **20**, 405-439 (1976).

21 Ikeda, K., Ikeda, T., Morikawa, K. & Kamiya, R. Axonemal localization of Chlamydomonas PACRG, a homologue of the human Parkin-coregulated gene product. *Cell Motil Cytoskeleton* **64**, 814-821 (2007). https://doi.org:10.1002/cm.20225

22 Bower, R. *et al.* DRC2/CCDC65 is a central hub for assembly of the nexin-dynein regulatory complex and other regulators of ciliary and flagellar motility. *Mol Biol Cell* **29**, 137-153 (2018). https://doi.org:10.1091/mbc.E17-08-0510
