# Supplementary material for: Native doublet microtubules from Tetrahymena thermophila reveal the importance of outer junction proteins
Source: Nat Commun. 2023 Apr 15;14:2168. doi: 10.1038/s41467-023-37868-0 (PMC10105768; doi:10.1038/s41467-023-37868-0)
Supplement: Supplementary file 3 — Description of additional supplementary files [file 41467_2023_37868_MOESM3_ESM.pdf]

Description of additional supplementary files

Supplementary Movie 1 : Highlighting the structure of the native DMT from Tetrahymena cilia.

Supplementary Movie 2 : An overview of the outer junction of the DMT form Tetrahymena cilia and their interactions.
